# Public Concerns during the COVID-19 Lockdown: A Multicultural Cross-Sectional Study among Internet Survey Respondents in Three Countries

**DOI:** 10.3390/jcm10081577

**Published:** 2021-04-08

**Authors:** Alona Emodi-Perlman, Ilana Eli, Nir Uziel, Joanna Smardz, Anahat Khehra, Efrat Gilon, Gniewko Wieckiewicz, Liran Levin, Mieszko Wieckiewicz

**Affiliations:** 1 Department of Oral Rehabilitation, The Maurice and Gabriella School of Dental Medicine, Tel Aviv University, Tel Aviv 6139001, Israel; dr.emodi@gmail.com (A.E.-P.); elilana@tauex.tau.ac.il (I.E.); niruziel@gmail.com (N.U.); gilon.efrat@gmail.com (E.G.); 2 Department of Experimental Dentistry, Wroclaw Medical University, 50-425 Wroclaw, Poland; joannasmardz1@gmail.com; 3 Faculty of Medicine and Dentistry, University of Alberta, Edmonton, AB T6G 2R3, Canada; anahat@ualberta.ca (A.K.); liran@ualberta.ca (L.L.); 4 Chair and Clinical Department of Psychiatry, Medical University of Silesia, 40-055 Katowice, Poland; gniewkowieckiewicz@gmail.com

**Keywords:** COVID-19, SARS-CoV-2, coronavirus pandemic, anxiety, depression, mental health

## Abstract

(1) Background: this study aimed to evaluate the worries, anxiety, and depression in the public during the initial coronavirus disease 2019 (COVID-19) pandemic lockdown in three culturally different groups of internet survey respondents: Middle Eastern (Israel), European (Poland), and North American (Canada). (2) Methods: a cross-sectional online survey was conducted in the mentioned countries during the lockdown periods. The survey included a demographic questionnaire, a questionnaire on personal concerns, and the Patient Health Questionnaire-4 (PHQ-4). A total of 2207 people successfully completed the survey. (3) Results: Polish respondents were the most concerned about being infected. Canadian respondents worried the most about their finances, relations with relatives and friends, and both physical and mental health. Polish respondents worried the least about their physical health, and Israeli respondents worried the least about their mental health and relations with relatives and friends. Canadian respondents obtained the highest score in the PHQ-4, while the scores of Israeli respondents were the lowest. (4) Conclusions: various factors should be considered while formulating appropriate solutions in emergency circumstances such as a pandemic. Understanding these factors will aid in the development of strategies to mitigate the adverse effects of stress, social isolation, and uncertainty on the well-being and mental health of culturally different societies.

## 1. Introduction

Coronavirus disease 2019 (COVID-19) is a novel severe respiratory syndrome caused by a new betacoronavirus, severe acute respiratory syndrome coronavirus 2 (SARS-CoV-2) [1,2,3,4,5]. The COVID-19 pandemic has caught the world by surprise. Within a relatively short time, most countries were affected and responded with partial-to-total lockdowns to curb disease spread [6]. The daily lives of people were greatly affected in every aspect, including restrictions in socializing, working (up to full quarantine), and/or planning for the future.

Mid-March 2020, the rate of contraction and the rate of deaths by COVID-19 constantly rose. On 15 March 2020, the number of daily confirmed cases in Israel was 2 per million people. Within one month (on 15 April 2020), the number rose to 45.32 per million (relative change of +2169%). The total number of confirmed deaths from COVID-19 during that period (15 March till 15 April) rose from 1 to 139 (relative change of +13,800%) [7]. On 19 March 2020, the Israeli government declared an almost complete lockdown. All schools, kindergartens, and universities were shut down, and schooling was continued (partially) through the internet. Leaving home to a distance greater than 100 m was prohibited except in the cases of emergency, shopping for basic products, or work in vital posts (specifically defined by the government). Most adults were either put on a no-pay leave or were instructed to work from home. Personal contact with non-cohabitating family members and/or with friends was prohibited, even during traditional religious family gatherings such as Passover.

The situation in Poland on 15 March 2020 was that of 0.41 per million COVID-19 confirmed cases. Within one month, the number rose to 8.97 per million (relative change of +2100%). The total number of confirmed deaths from COVID-19 during that period rose from 3 to 286 (relative change of +9433%) [7]. An almost complete lockdown was implemented in the country in mid-March, with regulations similar to those in Israel except for no limitation on the distance from home.

In Canada, the number of daily confirmed cases on 15 March 2020 was 0.71 per million people. Within one month (on 15 April 2020), the number rose to 34.32 per million (relative change of +4725%). During that period of time, the total number of confirmed death cases due to COVID-19 rose from 1 to 1008 (relative change of +100,700%) [7]. In the Province of Alberta, a state of public health emergency was declared on 17 March 2020. Schools, shops, arenas, restaurants, places of worship, recreation centers, bars, etc. were closed. All the other provinces across Canada declared a similar state of public health emergency.

Undoubtedly, all communities experienced feelings of separation, apprehension, stress, anxiety, and even depression. Uncertainty regarding the length of the situation and its final consequences only added to the anguish.

A recent review on the psychological impact of quarantine reported that it had substantial negative psychological effects on people, contributing to posttraumatic stress symptoms, confusion, and anger. Stressors included longer durations of quarantine, infection fears, frustration, boredom, inadequate supplies, insufficient information, financial loss, and stigma [8].

A number of studies have been published about the emotional aspects of the COVID-19 pandemic. For instance, Germani et al. described the impact of this pandemic on young Italian adults [9]. In a study conducted in Denmark, the researchers reported that the psychological well-being of subjects was negatively affected by the situation [10]. In a study performed in China, more than half of the participants showed a significant psychological disturbance due to the pandemic, while in a study from the United States, nearly half of the participants were found to be anxious [11,12]. In a study conducted in India, Varshney et al. reported that the factors predicting higher psychological impact among the general public were younger age, female sex, and presence of known physical comorbidity [13]. The infection might also have a putative tropism toward the central nervous system (CNS) that may explain some of the symptoms seen in clinical practice, with possible late neuropsychiatric manifestations [14]. Clearly, the pandemic took its toll on societies worldwide. The effect may vary among cultures and societies. A previous study showed that the COVID-19 pandemic caused significant adverse effects on the psycho-emotional status of both Israeli and Polish populations, resulting in the intensification of their orofacial pain. However, the two populations varied in their reaction to the stress. For example, the odds of occurrence of orofacial pain symptoms among Polish subjects were on average over 3 times higher than that among Israeli subjects [15]. Understanding the factors that cause worries, anxiety, and depression among different communities will enable us to develop response strategies to mitigate the adverse effects of stress, social isolation, and uncertainty on well-being, and physical and mental health in culturally different societies.

The present study aimed to evaluate the worries, anxiety, and depression in the public during the initial pandemic lockdown in three culturally different groups of internet survey respondents: Middle Eastern (Israel), European (Poland), and North American (Canada).

## 2. Materials and Methods

A cross-sectional online survey was conducted using anonymous questionnaires. The final questionnaire was compiled from a tool commonly used with regard to anxiety and depression (Patient Health Questionnaie-4 (PHQ-4), as detailed below), and specific questions referring to demographics and concerns specific to the COVID-19. The latter were agreed upon and tested for content validity by a group of subject matter experts (SMEs). The group consisted of four researchers (A.E.-P., I.E., N.U., and E.G.) who work at the Tel Aviv University and have vast academic experience in population studies. Each SMEs proposed questions for the study, and following discussions, the final questions were agreed upon. The questionnaire was compiled in Hebrew and translated to Polish by the Polish group. One of the Israeli researchers (I.E.), who is native in the Polish language, verified the Polish translation by retranslating it to Hebrew and vice versa. The Israeli group translated the questionnaire to English. One of the members of the Canadian group (L.L.), who is native in Hebrew, verified the English translation as above.

SurveyGizmo (www.surveygizmo.com (accessed on 19 April 2020)) was used to collect data. The survey was anonymous. Therefore, due to the need to avoid respondents’ identification, and according to the EU regulations, quality control measures could not be practiced.

In Israel, the survey was posted in Hebrew, which assured that only Hebrew-speaking Israeli responders participated. In Poland, the survey was posted in Polish, enabling the participation of only Polish-speaking participants. In Canada, the survey was posted in English, with most of the responders coming from the province of Alberta.

The surveys were posted at least 4 weeks after the implementation of the first lockdown in each of the countries while the pandemic was still progressing at a rapid pace (see below).

In Israel, the survey was posted on 16 April 2020. Since the beginning of the pandemic, the relative change in cumulative confirmed COVID-19 cases per million in Israel at that time was +1,294,700% [7]. The questionnaire was distributed through social media (WhatsApp groups and Facebook groups).

In Canada, collection of data started on 13 May 2020. The relative change in cumulative confirmed cases per million in Canada at that time was +7,356,700% [7]. The questionnaire was distributed through social media (Facebook, Instagram, Reddit, and WhatsApp groups).

In Poland, collection of data started on 29 April 2020. The relative change in the number of cumulative confirmed cases per million in Poland at that time was +1,263,900% [7]. The questionnaire was posted on Reddit (r/Polska subreddit).

All responses were obtained anonymously in all three countries.

The study was conducted in full accordance with the World Medical Association Declaration of Helsinki. In Israel, the Ethics Committee of the Tel Aviv University approved all the study procedures (ID: 0001332-1). In Poland, the study was approved by the Bioethical Committee of the Wroclaw Medical University (ID: KB-302/2020). In Canada, the Research Ethics Board at the University of Alberta approved the study (ID: Pro00100768). Informed consent was obtained from all the subjects as required.

### 2.1. Instruments

The following data were collected through questionnaires:

#### 2.1.1. Demographic and General Information

This included the following:

- Consent to participate in the study
- Gender
- Age—the age groups of participants were defined according to “young adults” (age of 18–35 years) and “adults” (36–56 years old) as accepted in the literature [16]. Subjects over 56 years old were defined as “older”.

#### 2.1.2. Personal Concerns Regarding the COVID-19 Pandemic

Subjects were requested to indicate the following:

- Whether they were feeling at risk of contamination by the virus (yes/no)
- To what extent does the pandemic make them worry about finances (5-score scale, ranging from 1—not at all to 5—very worried)
- To what extent does the pandemic make them worry about their physical health, namely, do they worry that the pandemic might negatively affect their physical status, including decrease in stamina, possible aggravation of prior systemic conditions, etc. (scale as above)
- To what extent does the pandemic make them worry about their mental health (scale as above)
- To what extent does the pandemic make them worry about their relations with meaningful figures in their lives, such as spouse, children, relatives, friends, and colleagues (scale as above)

#### 2.1.3. Information on Anxiety and Depression

Subjects responded to the Patient Health Questionnaire-4 (PHQ-4), a brief screening tool used for assessing anxiety and depression. The questionnaire is a four-item inventory and is rated on a 4-point Likert-type scale. It allows for a very brief and accurate measurement of depression and anxiety. The reliability and validity of PHQ-4 have been repeatedly established [17,18]. It has been incorporated as a part of various diagnostic protocols and has been translated to numerous languages, including Hebrew and Polish [19].

The total score of the PHQ-4 ranges from 0 to 12: the higher the score, the higher the chances of the presence of anxiety and depression. The recommended PHQ-4 cutoff scores are as follows: 0–2, normal; 3–5, mild; 6–8, moderate; and 9–12, severe [20]. In the present study, a total PHQ-4 score was used for comparisons among countries, genders, and age groups.

The questionnaire also enables separate assessments of anxiety and depression.

A score of ≥3 for the first two questions suggests anxiety. A score of ≥3 for the two last questions suggests depression. In the present study, scores of the first two questions (anxiety) and of the last two questions (depression) were used for comparisons among countries, gender, and age groups.

The questionnaire referred to the last 2 weeks, namely, to the lockdown period.

### 2.2. Statistical Analysis

Data were analyzed using the SPSS software.

A logistic regression analysis was carried out to evaluate subjects’ feelings of being at risk of contamination by the virus. Variables that were entered into the equation were country, gender, age, and the interactions among them (age × country, country × gender, age × gender, and age × country × gender).

Four 3-way analyses of variance (ANOVA) were carried out to analyze subjects’ worries regarding finances, physical health, mental health, and relations with others. The analyses evaluated the effects of country (×3), gender (×2), age (×2), and possible interactions among them.

Three additional 3-way ANOVA were carried out to analyze subjects’ total PHQ-4 score as well as the separate scores of anxiety and of depression. The analyses evaluated the effects of country (×3), gender (×2), age (×2), and possible interactions among them.

## 3. Results

In Israel, a total of 867 subjects responded to the questionnaire during the study period, out of whom 80.7% (*n* = 699) completed the questionnaire in full.

In Poland, a total of 1096 subjects responded to the questionnaire during the study period, out of whom 99.63% completed the questionnaire in full (*n* = 1092).

In Canada, a total of 548 subjects responded to the questionnaire during the study period, out of whom 75.9% completed the questionnaire in full (*n* = 416). Among the responders, 93% were Canadians, mostly from the province of Alberta (78.4%).

### 3.1. Demographics

The distributions of gender and age of the study participants are presented in Table 1.

Significant differences were observed among countries with respect to gender (*p* < 0.000) and age (*p* < 0.0000).

In all three countries, there was a higher proportion of female responders than males, with the number of female responders being the highest in Canada (83%) compared to Poland (58%) and Israel (66%).

In general, the Polish and Canadian participants were younger (76% and 83% in the “young adult” group, respectively) compared to Israeli participants, in which only 30% of participants were in this age group. Due to the small number of respondents in the “older” group (>56 years) in Poland and Canada (2.8% and 3.4%, respectively), this group was collapsed with the “adult” age group and only two age groups were analyzed: the “younger group” (18–35 years) and the “older group” (>35 years).

### 3.2. Personal Concerns Regarding the COVID-19 Pandemic

#### 3.2.1. Feeling at Risk of Contamination

Significant differences were found among the respondents with regard to concerns of being at risk of contamination by the virus with respect to country, gender, and age group. Poles felt most at risk of contamination (46.8% positive response) compared to Canadians (21.9% positive response) and Israelis (20.7% positive response).

Logistic regression (with the variables country; gender; age group; and the interactions age × country, country × gender, age × gender, and age × country × gender) showed that, compared to Israelis, the odds of Poles feeling at risk of contamination were over three times as much (odds ratio 3.2, 95% CI 2.37–4.32, *p* < 0.000). In the case of Canadians, the odds of feeling at risk of contamination were almost twice that compared to the Israelis (odds ratio 1.91, 95% CI 1.09–3.35, *p* < 0.05).

Older responders felt in general more at risk than the younger responders (31.4% versus 19.9% positive response, odds ratio 1.87, 95% CI 1.49–2.34, *p* < 0.000).

Women felt in general more at risk than men (35.8% versus 30.7% positive response). The odds of Polish women feeling at risk of contamination was 1.86 compared to Israeli women (95% CI 1.44–2.38, *p* < 0.000).

#### 3.2.2. Worry about Finances

Three-way ANOVA that compared subjects’ levels of worry about finances showed the main effects of country (F_(2,2173)_ = 8.53, *p* < 0.000; Israel < Poland < Canada), gender (F_(1,2173)_ = 4.74, *p* < 0.05; male < female), and age (F_(1,2173)_ = 6.33, *p* < 0.05; younger < older).

Significant interactions among the variables country, age, and gender were also observed (F_(2,2173)_ = 6.59, *p* < 0.005). Parameter estimates of the 3rd-order interactions were as follows: (i) country (IL) × gender (F) × age (young)—B = 1.12, 95% CI 0.33–1.90, *p* < 0.005; (ii) country (PL) × gender (F) × age (young)—B = 1.41, 95% CI 0.64–2.17, *p* < 0.000 (*IL* = *Israel*, *PL* = *Poland*, *F* = *female*).

Generally, Poles worried the least (M = 2.38) while Canadians worried the most (M = 2.84). Females worried more than males, and younger subjects worried more than the older age groups. The group that worried the least about finances was Polish males (both age groups, M = 2.27), while the group that worried the most was Canadian younger males (M = 2.33).

#### 3.2.3. Worry about Physical Health

Three-way ANOVA that compared subjects’ worries about their physical health showed the main effects of country (F_(2,2167)_ = 8.38, *p* < 0.000; Poland < Israel < Canada) and gender (F_(1,2167)_ = 4.44, *p* < 0.05; male < female). The effect of age was borderline (F_(1,2167)_ = 3.739, *p* = 0.05).

A significant interaction between gender and age was also observed (F_(2,2167)_ = 9.18, *p* < 0.005). Parameter estimates of the 2nd-order interaction were B = −0.721, *p* = 0.02, 95% CI lower bound −1.317, upper bound −0.124.

Generally, Poles worried the least (M = 2.26) while Canadians worried the most (M = 2.61) about their physical health. Females worried more than males. The group that worried the most about their physical health were Canadian older males (M = 2.91), while Israeli adult males showed the least concern (2.10).

#### 3.2.4. Worry about Mental Health

Three-way ANOVA that compared subjects’ worries about their mental health showed the main effects of country (F_(2,2174)_ = 44.62, *p* < 0.000; Israel < Poland < Canada), gender (F_(1,2174)_ = 33.61, *p* < 0.000; male < female), and age (F_(1,2174)_ = 15.75, *p* < 0.000; older < younger). No interactions were detected among variables.

Generally, Canadians showed the highest concern about their mental health (M = 3.14) while Israelis showed the lowest concern regarding this aspect of the pandemic (M = 1.97). Females worried more than males and younger subjects, worried more than older subjects.

#### 3.2.5. Worry regarding Relations with Meaningful Figures

Three-way ANOVA that compared subjects’ worries about their relationships with meaningful figures in their lives showed the main effects of country (F_(2,2172)_ = 6.75, *p* < 0.001; Israel < Poland < Canada), gender (F_(1,2172)_ = 11.73, *p* < 0.001; male < female), and age (F_(1,2172)_ = 24.48, *p* < 0.000; older < younger). A significant interaction was observed between gender and age (F_(2,2172)_ = 8.05, *p* < 0.000). Parameter estimates of the 2nd-order interaction were B = −0.478, 95% CI lower bound −0.869, CI upper bound −0.086, *p* < 0.05.

Generally, the group that worried the most about their relations with relatives and friends was the Canadian subjects (M = 2.69) while Israelis was the group that worried the least (M = 1.94). Females worried more than males, while younger subjects worried more than older subjects. In the three countries, older females showed higher concern regarding relations with relatives and friends than their male counterparts (M = 2.10 versus 1.96).

### 3.3. Anxiety and Depression

#### 3.3.1. Total PHQ-4 Score

Three-way ANOVA of the total PHQ-4 score showed the main effects of country (F_(2,2174)_ = 27.103, *p* < 0.000; Israel < Poland < Canada), gender (F_(1,2174)_ = 38.85, *p* < 0.000; male < female), and age (F_(1,2174)_ = 16.59, *p* < 0.000; older < younger). No interactions were observed among the variables.

All three countries scored within the “mild” category of the total PHQ-4 score. In general, Israeli respondents showed the lowest PHQ-4 scores (M = 2.91) and Canadian respondents showed the highest scores (M = 4.94). Females showed higher scores than males (M = 4.37 vs. 3.27), and younger subjects showed higher scores than the older age group (M = 4.43 vs. 3.24).

#### 3.3.2. Anxiety (According to PHQ-4)

Three-way ANOVA analysis of the separate anxiety scores showed a basically similar pattern for the main effects of country (F_(2,2173)_ = 16.418, *p* < 0.000; Israel < Poland < Canada), gender (F_(1,2173)_ = 46.181, *p* < 0.000; male < female), and age (F_(1,2173)_ = 10.843, *p* < 0.000; older < younger). No interactions were observed among variables.

None of the countries reached a definite score defined of “anxiety” (>3). Nevertheless, Israelis were the least anxious group (M = 1.46) while Canadians were the most anxious one (M = 2.65). Females were more anxious than males (M = 2.22 vs. 1.42), and younger subjects were more anxious than the older age group (M = 2.14 vs. 1.61).

#### 3.3.3. Depression (According to PHQ-4)

A similar pattern was also revealed for the separate assessment of depression. Three-way ANOVA of the separate depression scores showed the main effects of country (F_(2,2174_) = 49.00, *p* < 0.000; Israel < Poland < Canada), gender (F_(1,2174)_ = 29.94, *p* < 0.000; male < female), and age (F_(1,2174)_ = 18.70, *p* < 0.000; older < younger). No interactions were observed among variables.

None of the countries reached a score definition of “depression” (>3). Nevertheless, Israelis were the least depressed group (M = 1.14) while Canadians were the most depressed one (M = 2.31). Females were more depressed than males (M = 2.01 vs. 1.54), and younger subjects were more depressed than the older age group (M = 2.12 vs. 1.40).

## 4. Discussion

In late December 2019, a new unfamiliar public health threat, the COVID-19 pandemic, began to spread around the world. With almost complete uncertainty concerning the method of virus spread and the modes of treatment, insufficient availability of local health services, and lack of a vaccine or efficient drugs for treatment, most countries adopted the policies of social distancing and partial-to-total lockdown. The new situation burdened people not only with immediate severe health threats but also with economic uncertainty and social isolation, causing further potential deleterious effects on their mental and physical health.

From recent studies indicating the direct and indirect influences of the pandemic, it is apparent that subjects around the world might react differently to the new stressful situation. In the present study, subjects from different regions—Middle Eastern, European, and North American—were compared in terms of their personal worries and emotional reactions. A summary of the main effects of the study is presented in Table 2.

The study showed that subjects’ reactions to the situation varied significantly among the three countries. Except for worry about physical health, Canadians appeared to be the most worried. Israelis appeared to be consistently less worried than the Canadians, while the Poles showed mixed behavior.

Israelis are used to changing their routine way of life within a short period as they frequently face sudden emergencies. The country is under constant security threats from the outside (at the borders) and the inside (terror attacks). Every few years, an emergency arises requiring the citizens to react quickly (i.e., stay in shelters). In general, the armed forces are highly trusted to contain the situation and to bring back normalcy. During the initial stage of the COVID-19 pandemic, the country reacted quickly. Borders were closed, and the army was mobilized to help. Although the political system underwent continuous changes (despite several rounds of indecisive elections, there was great difficulty in creating a stable government), the pandemic situation was generally under control.

Unlike Israel, Canada has not faced any major emergencies for many decades. Although the country was affected by severe acute respiratory syndrome (SARS) in 2003, which led to partial quarantine, the effects were evident mainly in Ontario [21]. Based on the SARS experience, one could assume that Canadians were more prepared for the COVID-19 pandemic and, therefore, less worried about its different aspects compared to other countries such as Poland, which had no such experience. Interestingly, the results were found to be quite the opposite. The study showed that Canadians worried more than the two other groups regarding their finances, physical health, mental health, and relations with relatives and friends.

Apparently, the unexpected threat rocked the lives of the Canadian people to a greater extent than the other two countries. The results were in line with a previous publication regarding the impact of COVID-19 in Canada, which showed that 87% of the population were concerned about the impact on vulnerable people, 21% about their own health, 36% about family stress from confinement, and 34% about maintaining social ties [22].

During the initial stage of the pandemic in Poland, a public opinion study showed that most of Polish citizens were concerned about their own health and about the health of their relatives, especially those of old age [23]. A previously published survey also showed that up to 57% of Polish citizens had concerns about their finances due to the COVID-19 pandemic [24]. The present study confirmed these results to some extent as Polish participants showed the second highest concern about mental health and relations with relatives and friends.

Among the three studied countries, one of the most prominent differences was observed for the subjects’ perceptions of being at risk of virus contamination. The odds of Poles and Canadian to worry about this issue were two to three fold higher than those of the Israelis. This may be explained by the advanced and generally good public health services available in Israel. In the country, all citizens have governmental health insurance and are entitled to all the necessary health services with no additional costs (besides a mandatory monthly fee). Hospitals are required to maintain high medical standards, and medical personnel are well-trained. Up until now, Israel has been efficiently carrying out vaccination processes against COVID-19, with high percentages of the adult population being already successfully vaccinated.

Although healthcare in Poland does not differ significantly from that in Israel and medical costs are reimbursed to some extent, access to some specialist procedures is limited. Due to the extended wait time for their implementation, there is distrust among Poles about their national healthcare system.

Apparently, the differences among countries with regard to subjects’ worries about the situation are also reflected in their emotional status (anxiety and depression).

In a recent Canadian survey series, performed during the COVID-19 pandemic only half of the Canadians reported excellent and very good mental health [25]. In Poland, the ranges of depressive, anxiety, and stress symptoms were around 50% during the COVID-19 outbreak [26]. In Israel, more than one-third of the responders reported stress and anxiety due to the crisis [27].

The impact of COVID-19 lockdowns on the mental health of people suffering from psychiatric disorders may be even more substantial and may lead to an increase in general psychopathology, anxiety, fear, and stress related to the quarantine. Tele-psychiatry was implemented in some countries to address population concerns and worries. Acceptance of telemedicine by decision makers might allow for quick response in times where face-to-face visits are not accessible [28,29]. As the pandemic still prevails around the globe, such an approach should be seriously considered.

The differences between genders, in levels of worries, anxiety, and depression, are not surprising. As a global phenomenon, women seem to be hardest hit by unemployment due to the pandemic [30,31,32,33]. Regretfully, information regarding domestic and professional variables such as family responsibilities, education level, employment type, or annual income was not collected in the present study. According to the United Nation policy brief, the pandemic amplified and heightened all preexisting inequalities (such as gender inequality) and exposed vulnerabilities in social, political, and economic systems, which in turn amplify the impacts of the pandemic [34].

The effect of age on the personal concerns and emotional aspects of subjects reported in the present study was inconsistent. Findlay et al. showed that Canadian youth had a higher risk for poor mental health both before and during the COVID-19 pandemic, while Okruszek et al. showed that young Polish adults were more concerned about the collapse of healthcare than any other issue [35,36,37]. This confirms that, in the days of health threats and financial uncertainty, anxiety, depression, and worries about mental health and social relations affect young adults worldwide. It is noteworthy that the group of responders over the age of 56 years in the present study was especially small in both Canada and Poland and therefore could not be properly analyzed. Possibly, some of the subjects at that age are not active in the social media used to distribute the questionnaire.

The results showed complex interactions among some of the study variables. Men and women belonging to different age groups reacted differently in each of the examined countries. These interactions suggest that the reactions of different subgroups (gender/age) are affected by subtle factors that are specific for each country. Further research will be needed to better analyze these differences.

Personal concerns evoked by the pandemic not only adversely affect the emotional status of subjects but also can also take a toll on physiological phenomena [37,38,39]. A previous study showed that personal worries, depression, and anxiety during the COVID-19 pandemic can act as predictors of symptoms that cause aggravation of chronic pain, the effect of which varies between communities [15]. Researchers, clinicians, and politicians are in need of literature that can help them understand the impact of this crisis on people’s emotional and mental health, which can be long lasting.

Study limitations: although an effort was made to perform the survey at similar times as far as the pandemic progression is concerned, the slightly later timing of gathering data in Canada might have biased the results in this country. Moreover, the study was carried out as an anonymous internet survey with no actual ability to control the participants. As a result, the study groups differ as far as gender and age are concerned and are not necessarily representative of the country’s populations. As pointed above, there was a under representation of the older age group (>56), which might have shown a different pattern of behavior (such as being more worried about contamination and physical health but possibly less worried about financial issues than the younger groups).

## 5. Conclusions

Better understanding the factors that cause worries, anxiety, and depression among different communities will enable us to develop response strategies to mitigate the adverse effects of stress, social isolation, and uncertainty on well-being, and physical and mental health in culturally different societies.

## Figures and Tables

**Table 1:** Gender and age distributions.

Country		Israel		Poland		Canada	
		No.	%	No.	%	No.	%
Gender	Female	465	66.5%	638	58.3%	343	82.7%
	Male	234	33.5%	457	41.7%	72	17.3%
Age	18–35	203	29.6%	830	75.8%	337	82.8%
	35–56	283	41.3%	234	21.4%	56	13.8%
	>56	199	29.1%	31	2.8%	14	3.4%

**Table 2:** Summary of the study main effects.

Variable *	Country **	Gender	Age
Feeling at risk of contamination	IL < CA < PL	Male < female	Younger < older
Worry about finances	PL < IL < CA	Male < female	Younger < older
Worry about physical health	PL < IL < CA	Male < female	
Worry about mental health	IL < PL < CA	Male < female	Older < younger
Worry about relations	IL < PL < CA	Male < female	Older < younger
PHQ-4-total, Anxiety, Depression	IL < PL < CA	Male < female	Older < younger

* Variables as defined in the text; ** country: IL—Israel, PL—Poland, CA—Canada; PHQ-4—Patient Health Questionnaire-4.

## Data Availability

The data presented in this study are available on reasonable request from the corresponding author.
